# Vascular Alterations and Sexual Function in Systemic Sclerosis

**DOI:** 10.1155/2010/139020

**Published:** 2010-08-05

**Authors:** Ann Julie Impens, James R. Seibold

**Affiliations:** ^1^Scleroderma Program, University of Michigan, 24 Frank Lloyd Wright Drive, Ann Arbor, MI 48106, USA; ^2^Division of Rheumatology, University of Connecticut Health Center, MARB MC 5353 Room N3020, 263 Farmington Avenue, Farmington, CT 06034, USA

## Abstract

Sexual dysfunction is common in systemic sclerosis (SSc). Male erectile dysfunction (MED) has been reported in around 80% of subjects and more than half of female patients fulfill criteria for diagnosis as female sexual arousal Disorder (FSAD). While some evidence supports a role for cavernosal fibrosis, abundant data suggest that MED is yet another clinical feature of SSc related to vasculopathy. The contribution of vasculopathy to the more complex issues of female sexual dysfunction is less clear. Inhibitors of Type V phosphodiesterase are effective in men with MED secondary to SSc. Limited study in women suggests inconsistent effects on behavior (frequency) but not on measures related to perfusion. Sexual activity is an important component of quality of life and an important domain for the caregiver to address; it is not clear that it warrants primary consideration as a consistent measure of scleroderma-related vasculopathy.

## 1. Introduction

Any comprehensive hypothesis concerning the pathogenesis of systemic sclerosis (SSc, scleroderma) must account for the varying contributions of vascular damage, extravascular tissue fibrosis, and inflammation [1]. While certain clinical features of SSc are overt expressions of vascular injury (renal crisis, pulmonary arterial hypertension, and Raynaud phenomenon), our understanding of other organ involvements is less well understood. The clinical and physiologic expressions of SSc on sexual function are one such example.

Complications from scleroderma do have a negative impact on sexual function and in turn on the overall quality of life. In spite of the 80% female predominance of SSc [1], most of the studies on the effect of SSc on sexual function have involved men [2–9]. Male erectile dysfunction (MED) has been noted in as many as 81% of afflicted persons [9]. MED in SSc can be reasonably attributed to scleroderma vasculopathy although cavernosal fibrosis has been implicated as well [10]. Little is known of the impact of scleroderma on female sexual functioning and on quality of female sex life. The general difficulties in researching female sexual function might explain some of the lack of interest in this area although there has been a marked change in the past year with the publication of several studies [11–13]. Daily and long-acting PDE-5 inhibitors have been proven to be safe and effective for MED in males with SSc [10]. To our knowledge, no studies have been published on successful pharmacological interventions in female SSc patients with sexual impairment.

## 2. Male Erectile Dysfunction

MED is defined as the consistent inability to attain and maintain an erection sufficient to permit satisfactory sexual performance [14]. The first report of an association between erectile dysfunction and SSc was as recently as 1981 [15]. Prevalence of MED in SSc has been found to range from 12% to 81% [9, 16]. The causes of ED associated with SSc are unclear although a number of factors have been suggested including vascular, fibrotic, and neuropathic/dysautonomic factors [10, 15, 17]. While some studies reported correlations between ED in SSc and testosterone and prolactin levels [15, 17] others have not [18–20]. Walker et al. [10] conclude that there is no support for a hormonal basis for ED in SSc as no consistent abnormalities in serum testosterone, follicle-stimulating hormone, luteinizing hormone, prolactin, estradiol, or thyroid hormones have been found. 

Several studies have ruled out a neurological basis for ED in SSc [17, 21]. Recent studies have found links between ED in SSc and penile blood pressure [22] and penile temperature [23], leading to a link between ED in SSc and vasculopathy. A strong connection between endothelial function and ED has been reported in the general population [24], supported by the strong linkage of MED with coronary artery disease. In this construct, endothelial injury and dysfunction result in diminished nitric oxide (NO) production. During normal penile erection, nitric oxide is released at the nerve endings of the penis. Endothelial cells are also a source of nitric oxide. Nitric oxide diffuses into the vascular smooth muscle cells in the penile corpus cavernosum stimulating guanylyl cyclase and production of cyclic guanosine monophosphate (cGMP). This in turn activates cGMP-dependent protein kinase (PKG), phosphorylation of several proteins, lowering of intracellular cell calcium, or sensitivity to calcium leading to muscle relaxation. This relaxation leads to an increase of blood in the corpus cavernosum leading to penile erection [25]. An insufficient release of nitric oxide from nerve endings or endothelium can lead to an attenuated production of cGMP. 

Penile vascular damage in SSc patients was assessed using Duplex ultrasonography [2, 23] showing that penile thermal abnormalities are present in almost all SSc patients. Penile fibrosis has also been shown to be present in almost all SSc patients. Nehra et al. [19] report severe veno-occlusive dysfunction based on histological analysis confirming severe corporeal fibrosis from a biopsy evaluation of a single SSc ED patient. They postulate that fibrosis could lead to veno-occlusion leading to a reduction of the trabecular fibroelastic compliance and trabecular smooth muscle tone which depend on neurogenic-and endothelial-dependent vasomotility [23]. Penile fibrosis has also been shown to occur in almost all SSc patients with the presence of thickening of the tunica albuginea and hyperechoic spots inside the corpera carnevosa [22]. Merla et al. conclude that their results showing fibrosis and thermoregulatory dysfunction in SSc patients with ED support the hypothesis that structural modifications induced by SSc lead to a reduced capability of heat exchange and vascular damage [23]. 

Phosphodiesterase type-5 (PDE5) is an enzyme which degrades cGMP. PDE-5 inhibitors enhance erectile function by blocking degradation of cGMP leading to an increase in intracellular cGMP in the corpus cavernosum and penile vasculature. The result is an increase in the relaxation of the smooth muscle leading to an increased blood flow and penile erection [25]. There are three PDE-5 inhibitor compounds licensed for treatment of MED: sildenafil, vardenafil, and tadalafil. These three compounds are similar in pharmacologic and clinical characteristics. Studies in the general population have shown these to be safe and generally effective depending on the etiology and severity of the erectile dysfunction [26]. MED in SSc has been less well-studied. Ostojic and Damjanov [21] described a small case series with unsatisfactory response to on demand sildenafil (25–50 mg). An open label randomized cross-over study by Aversa et al. [27] compared 20 mg tadalafil on demand versus 20 mg in a fixed alternate day schedule. On the fixed alternate day schedule, a significant improvement was reported for the flow-mediated dilatation, peak flow velocities of cavernous arteries, reduced plasma levels of ET-1 and vascular cell adhesion molecules (markers of endothelial function), and morning erections. No such results were found for the on-demand regimen suggesting that a constant plasma level of PDE-5I might be important for the treatment of MED in SSc [10]. Proietti et al. [2] report the results of a study with a daily 10 mg dose of tadalafil for 12 weeks in 14 SSc patients with varying degrees of ED. Improvements were found in erectile function, vascular measures of cavernous arteries, morning erections, and plasma ET-1 levels (reduced levels).

## 3. Female Sexual Dysfunction

There is an inequality in the number of studies focusing on male versus female sexual dysfunction (FSD) in the general population [28] and the same holds true in SSc although the disease primarily affects women. The complexity and multifactorial nature of female sexual response and female sexual dysfunction adds to the difficulty in research in this area. FSD can have psychological and social components in addition to medical and physiological components. FSD is defined as persistent or recurring decrease in sexual desire, persistent or recurring decrease in sexual arousal, dyspareunia, and a difficulty in or inability to achieve orgasm [29]. It is reported to affect 20%–50% of all women and can severely impact quality of life and interpersonal relationships [28, 29]. 

Changes associated with SSc can have a negative impact on female sexuality and sexual functioning. Symptoms such as skin tightening around the vaginal introitus, joint contractures, muscle weakness, changes in skin around the breasts and breast muscle, and joint pain have been found to be associated with lower levels of sexual functioning, desire, arousal, lubrication, and satisfaction [4, 6, 30]. Changes in the vaginal mucosal may lead to difficulties with lubrication. Many SSc patients experience significant limitations on exercise capacity with dyspnea, decreased stamina, and coughing that may interfere with certain sexual behaviors [4, 30]. Medications used to treat SSc-related symptom are also known to impact sexual desire and sexual functioning [30].

The number of women with SSc reporting sexual dysfunction is higher than those reported in the general population and also higher than those reported in studies on other chronic conditions [11–13]. Bhadauria et al. [30] reported that more than 50% of women with SSc had significantly fewer orgasms and that they reported a decrease in the intensity of their orgasms compared to fewer than 20% of RA patients. In a study by Sampaio-Barros et al. [31], 37% of sexually active SSc patients mentioned dyspareunia. Some female SSc patients are sexually inactive because of complications related to their disease. One study reported that 17% of subjects were sexually inactive because of issues caused by their disease [11]. 

While there are many studies on the use of PDE-5 inhibitors in the treatment of ED there are few studies on their use in the treatment of female sexual arousal problems and their effectiveness is in question [32]. Our understanding of the effects of these drugs on female sexual psychophysiology is limited. Animal models of female sexual response show a similar physiological effect of PDE-5 on vaginal and clitoral tissues compared to males with NO synthase (NOS) being active in the vaginal epithelium and PDE5 enzyme found in vaginal smooth muscle tissue and clitoral shaft. PDE5 inhibition with sidenafil in female rabbits have been shown to lead to increased smooth muscle relaxation. Chivers and Rosen [32] conclude that there is clear evidence for the role of NO neurotransmitter mechanisms in mediating blood flow and smooth muscle relaxation in the clitoral shaft, vaginal endothelium, and uterine tissues. However, PDE5 inhibitors are not found to be as efficacious in women with sexual dysfunction as in men with ED [32, 33]. Studies focusing on the physiological effects of PDE5 on genital vasocongestion consistently report effects on genital sexual response while those using self-reported measures provide mixed results. This is likely due to problems related to the classification and measurement of FSD in women and difficulties related to finding meaningful endpoints in clinical trials due to the complexity of female sexual response compared to men [32]. Chivers and Rosen [32] attribute this lack of efficacy of PDE-5 inhibitors in women to gender differences in the concordance between physiological and psychological components of sexual response. 

**Figure 1:**
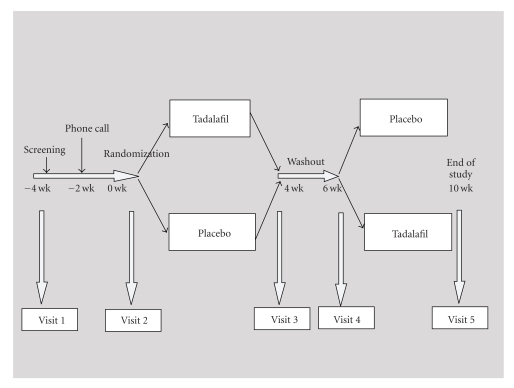
Study design.

While there are few studies on the effectiveness of PDE-5 inhibitors for FSD in the general population, no studies were found in SSc patients. The paper by Chivers and Rosen [32] regarding the effectiveness of PDE-5 inhibitors for FSD did not include any studies on the longer acting drug, tadalafil. As part of a study on the safety, tolerability and effectiveness of tadalafil for Raynaud phenomenon (RP) [34], female subjects were asked to attempt sex at least once weekly to evaluate potential effects on quality of female sexual functioning. This prospective randomized, double-blinded, placebo-controlled, and cross-over study compared oral tadalifil at a fixed 20 mg dose daily for a period of 4 weeks versus placebo in female SSc patients. Tadalafil was found to be well tolerated but no statistically significant differences were found in Raynaud Condition Score, frequency or duration of RP episodes between treatment periods. The study design can be found in Figure 1. There were a total of 5 visits: a screening visit (Visit 1), three intermediate visits (Visits 2–4), and an exit Visit. Data obtained specifically regarding sexual function included the female sexual function index (FSFI) and a sexual activity log including the frequency of sexual activity. This was obtained at Visit 2 (baseline) and Visits 3 and 5 (after treatment or no treatment period). The FSFI quantitatively assesses six domains of sexual functioning: desire, subjective arousal, lubrication, orgasm, pain, and satisfaction [35]. 

A total of 39 patients met all inclusion criteria, agreed to attempt sexual activity at least once weekly and successfully completed all 5 visits. When comparing change from baseline between drug and placebo on the six domains of the FSFI no statistically significant differences were found although patients reported greater change when on drug versus when on placebo. A significant difference was found between drug and placebo in the number of sexual activities with SSc patients reporting more frequent sexual activities when receiving drug than when on placebo (mean number sexual activity drug = 7.24 with sd = 4.53 and a mean of 6.12 and sd of 3.3 on placebo). This difference was statistically significant (*P* =  .021; 2-tailed) as was the change from baseline (1.49 and sd = 2.55 on drug and x =  .36 and sd = 2.16; *P* =  .024 2 tailed). The only other statistically significant difference in this study was found in a change from baseline in the sexual desire domain of the FSFI for drug although this was not statistically different from the change in the placebo group. 

Some of the more recent studies on female sexual functioning in SSc confirm the complexity of studying female sexual function as well as the psychological components of it. Depressive symptoms were found to be associated with impaired sexual functioning and sexual distress in SSc [11, 13]. In our own studies of quality of female sexual function, we found stronger correlations with the mental component of the SF-36 than with the physical component score [11]. One lesson is that health care professionals should not dismiss consideration of sexual care and advice in any patient with systemic sclerosis. 

## 4. Conclusions

Sexual dysfunction is a common problem in both men and women with systemic sclerosis. In males, the dominant issue is erectile dysfunction which in turn seems tightly linked to vascular dysfunction. Sexual dysfunction in the female patient is no less prevalent but is considerably more complex. Aspects of female sexual response thought to be vascularly influenced—clitoral engorgement, lubrication, orgasm—are but some of the components of quality of sexual life. To our knowledge, only one study with PDE5-I has been performed in women with confusing and small effects whereas PDE5-I are a mainstay agent in MED. The clinician should be reminded that women with scleroderma remain sexually active overall (60%) in spite of a host of physical and psychological difficulties associated with their disease. Complete care should consider agents for vaginal lubrication, advice about positioning and attention to sexual adverse events of therapies.
